# Social Organization Participation, Government Governance and the Equalization of Basic Public Services: Evidence from China

**DOI:** 10.3390/ijerph16162996

**Published:** 2019-08-20

**Authors:** Gangqiang Yang, Yongyu Xue, Yuxi Ma

**Affiliations:** 1 Institute for the Development of Central China, Wuhan University, Wuhan 430072, China; 2 Development Research Center of the Yangtze River Economic Belt, Wuhan University, Wuhan 430072, China

**Keywords:** social organization, government governance, equalization of basic public services

## Abstract

This paper uses the methods of System Generalized method of moments (SYS-GMM), mediation effect and linkage effect to investigate the relationship among social organization participation, government governance and the equalization of basic public services from 2007 to 2017 in China. The empirical results show that the participation of social organizations and improvement in the government governance can promote the equalization of basic public services. The government has a greater capacity to drive the equalization of basic public services, but the density of social organizations can serve as a mediator in the equalization of basic public services. The government governance and social organization density have a strong linkage effect, but the link with social organization quality is weak. Furthermore, a linkage effect is evident in medical and health care, public education, environmental protection, and public culture but not in public science and social welfare.

## 1. Introduction

Achieving the goal of equalizing basic public services is not only a requirement for improving the quality of life of residents but also an inevitable choice for the transformation from a labor-intensive economy to a capital and technology intensive economy in China. By the end of 2018, the penetration rate of compulsory education reached 94.2%, the participation rate of basic medical insurance exceeded 96%, the comprehensive coverage rate of radio and television reached 98%, and the construction of more than 60 million urban affordable housing projects had begun. China has established a national basic public services system that aims to cover all residents, but problems such as insufficient supply, low quality, and uneven development still exist [1]. Therefore, how to enable different departments to accelerate the equalization of basic public services has become an important aspect of solving the main social contradictions in China and the focal point of supply-side structural reform.

As the economy of China enters a new normal, the population structure, social structure and consumption structure show a new development trend, and residents’ demand for basic public services is increasingly diversified and complicated. Thus, the mode of government-led services has been unable to achieve an efficient supply [2] (p.4). Furthermore, the private supply mode may be selective, and “market failure” may affect the supply efficiency of basic public services.

The Public Governance Theory proposed multi-agent participation in public goods and service supply [3,4,5] and also provided theoretical support for the Third Sector, represented by social organizations, to participate in basic public services [6].The word “governance”, which does not represent official authorization, works effectively [7], so the theory of public governance advocates for weakening the sole authority of government [8] and reconstructing the relationship between the government and the market. Through network governance [9,10] and performance evaluation [11], the theory encourages interaction and cooperation among government, the market and the third sector. The Public Governance Theory has been regarded as a useful tool to solve for “government failure “and “market failure”. Lester M. Salamon’s pioneering research investigating the view of “third-party governance” divides the responsibilities of the government and nonprofit sectors [12]. As long as there is a clear definition of property rights and interest evaluation [13,14], social organizations can change the relationship between citizens and government [15] as well as play an important role in infrastructure, environmental protection, community welfare and even public safety [16,17,18,19].

Social organizations in China have gradually become an important tool for meeting the needs of residents’ individualized and specialized interests, especially in the fields of medical and health care, public education, and cultural construction [20,21,22,23,24]. By the end of 2017, the number of registered social organizations in the country had reached 762,000; the number of employees in social organizations totaled 8.647 million. The number of social organizations and employees in the three types of social organizations has shown an upward trend, and the value added by social organization services has increased from 30.7 billion to 65 billion over the last decade.

However, in contrast to social organizations in Western countries, which have a high degree of autonomy, the accelerated development of social organizations in China is the result of the government’s “weaken authoritarianism” measures, and social organizations typically have weak discourse power and strong dependency. Some practical dilemmas, such as an insufficient ability to regulate resources and a low degree of specialization, have led to their extensive development within the narrow living space given by the government [25,26]. Social organizations are still in an early stage of development and are characterized by an uneven geographical distribution and industrial relevance. Although there is consensus that the quality of social organizations directly affects the efficiency of public services [27], the problem of covering a small population and a small service area is more prominent. At this stage, increasing the number of social organizations and their area of coverage may be an effective way to promote the equalization of basic public services in China [28]. Therefore, we propose the following research hypothesis:

**Hypothesis 1**: *Currently, social organizations have a weak driving capacity to promote the equalization of basic public services, while the density and quality of social organizations are heterogeneous. Improving the coverage of social organizations is a top priority for promoting the equalization of basic public services in China*.

Although the role of social organizations has become increasingly prominent, the provision of basic public services remains a basic function of the government and a logical starting point of state governance [29]. It is generally believed that the decline in government governance could significantly reduce the quantity and quality of public service provision [30,31,32,33].

In China, there is an asymmetric dependence between social organizations and the government. The government tends to intervene in social organizations through certain institutional arrangements to control social organizations [34]. First, the government controls and provides guidance [35] for social organizations at the level of social cognition and activity through registration, annual inspection, evaluation and law enforcement; second, the basic laws formulated by the government determine the basis for solving disputes and contradictions in social organizations; third, the main ways in which social organizations participate in the supply of basic public services involve cooperation with the government. Direct government purchase and financial subsidies constitute the main sources of funds for the operation of social organizations.

Furthermore, the relationship between the government and social organizations is a microcosm of the relationship between the state and society in the governance of public affairs. With the continuous transformation of government functions, the linkage effect of the government governance and social organization participation in promoting the equalization of basic public services has become increasingly prominent. Through a clear reward and punishment mechanism [36], supervision [37], a moderate management system [38] and an efficient partnership [39] resulting in synergies [40], the efficiency and quality of the supply of basic public services can be significantly improved [41,42]. The development of social organizations cannot be separated from the support and help of the government, and an important responsibility of social organizations is to undertake some public service functions of the government. Thus, an analysis of the mediation and linkage effects between the government governance and social organization participation is necessary in studying the equalization of basic public services.

Additionally, based on the analysis of hypothesis 1, heterogeneity exists in social organization density and quality, which affects the equalization of basic public services. An important reason for the heterogeneity is the different degrees of government guidance regarding the quantity and quality of social organizations. Therefore, the linkage between the government governance capacity and social organization density and quality has heterogeneous effects. The manifestation of this heterogeneity lies in the difference of the linkage effect between the government governance and the participation of social organizations in different types of basic public services. In summary, we present the following research hypothesis:

**Hypothesis 2**: *The participation of social organizations and the government governance have mediation and linkage effects on the development of basic public services. However, government governance has a heterogeneous effect on social organization density and quality, and different types of basic public services require different provider characteristics*.

Based on the two hypotheses, this paper uses System Generalized method of moments (SYS-GMM) to analyze the relationship among social organization participation, government governance capacity and the equalization of basic public services. Then, we examine how social organization participation and government governance capacity influence the equalization of basic public services by using the methods of mediation and linkage effects and investigate the heterogeneity in different basic public services. Finally, this paper provides practical guidance for promoting the equalization of basic public services from the perspectives of social organizations and the government.

The remainder of this paper is organized as follows. The second part introduces the models, variables and data sources; the third part presents the empirical analysis; the fourth part discusses the robustness of the empirical results and the fifth part concludes the paper.

## 2. Materials and Methods

### 2.1. Main Variables

#### 2.1.1. Dependent Variable

The dependent variable in this paper is the equalization of basic public services. The equalization of basic public services refers to the basic rights of residents to enjoy basic public services at roughly the same level. Equalization does not emphasize that all residents enjoy the same basic public services, but on the premise of recognizing the differences between regions, urban and rural areas, and ensuring that residents enjoy basic public services above certain standards [43].

There are two perspectives to measure the equalization of basic public services: Input and Output. When considering the input perspective, scholars often choose indicators such as per capita financial budget and per capita expenditures to measure the equalization of public services [44,45]. For the output perspective, scholars mostly use the entropy method to construct a comprehensive evaluation system based on multiple public service projects [46].

In this article, we measure the equalization of basic public services from the output perspective, and the definition of equalization adopted in the documents of “Notice on Promoting the Equalization Plan of Basic Public Services During the 13th Five-Year Plan” and “The 12th Five-Year Plan of Basic Public Service System” [1,47]. We do not consider the categories in which social organizations cannot participate (Basic Social Insurance, Basic Housing, Employment and Entrepreneurship) and for which data are limited (Disability Protection). At the same time, we include in our research Public Science [48] and Environmental Protection [49], which belong to basic public services and have been studied by large number of studies [50,51]. Thus, this paper divides basic public services into the following six categories: Public Education, Medical and Health Care, Public Culture, Public Science, Environmental Protection, and Social Welfare. Seventeen indicators are selected to build an index of basic public services equalization.

We use the entropy method, which can avoid subjectivity interference in the construction of an evaluation index, to determine the weight, followed by the weighted summation all the variables to obtain our index system on the equalization of basic public services. The calculation process of the basic entropy method is set as (1)–(4).

The statistical value of each indicator is set to $\;r_{ij}^{\prime }$. To eliminate the influence of different units among the indicators, the extreme value method is first used to standardize the data, and the standardized matrix is $\;R_{ij}$ as follows:

(1) $$R_{ij}=\frac{r_{ij}^{\prime }-\underset{j}{\min}\left|r_{ij}^{\prime }\right|}{\underset{j}{\max}\left|r_{ij}^{\prime }\right|-\underset{j}{\min}\left|r_{ij}^{\prime }\right|}$$

We then calculate the information entropy of each indicator as follows:

(2) $$\;H_{i}=-k\overset{n}{\underset{j}{\sum }}f_{ij}\ln f_{ij}$$

In addition,

(3) $$\;f_{ij}\;=\frac{R_{ij}}{\sum_{j=1}^{n}R_{ij}},k=\frac{1}{\ln n}\left(if\;f_{ij}=0,f_{ij}\ln f_{ij}=0\right)$$

After the entropy information is obtained, the entropy value of each indicator can be determined according to the following formula [52]:

(4) $$w_{i}=\frac{1-H_{i}}{m-\sum_{i=1}^{m}H_{i}}$$

The dependent variables are shown in Table 1.

#### 2.1.2. Independent Variables and Control Variables

The key independent variables in this paper are social organization participation and the government governance. As the welfare state shrinks, the third sector represented by social organizations aim to fill the void and meet unsatisfied social needs and alleviate the financial pressure faced by the government to provide public services [53,54]. However, funds and categories for the participation of social organizations in public services, to various degrees, depend on the extent of devolved power, which is required for government governance [55]. In addition, supervision from government is an effective means of preventing social organizations from becoming profit-making. Therefore, exploring social organization participation and government governance is necessary to study the development of basic public services.

Social organization is an administrative term that mainly includes private non-enterprises, social groups and foundations in China. It has the similar concept and characteristics to the Third Sector, NGO or NPO. There are still many organizations that belong to the Third Sector in concept but that do not belong to social organizations in China, such as public institutions that are controlled by the government and grassroots NGOs that are not registered. In this paper, we use the term “social organizations” not only to show its uniqueness, but also to consider the selection variables. Social organization density and quality are selected to construct an index of social organization participation. The density of social organizations is represented by the number of social organizations per 10,000 people, while the quality of social organizations is measured by the proportion of employees with undergraduate education to all employees in social organizations. The entropy method was used to calculate the weight and then the weighted summation all of the variables to obtain the index system.

**Figure 1:**
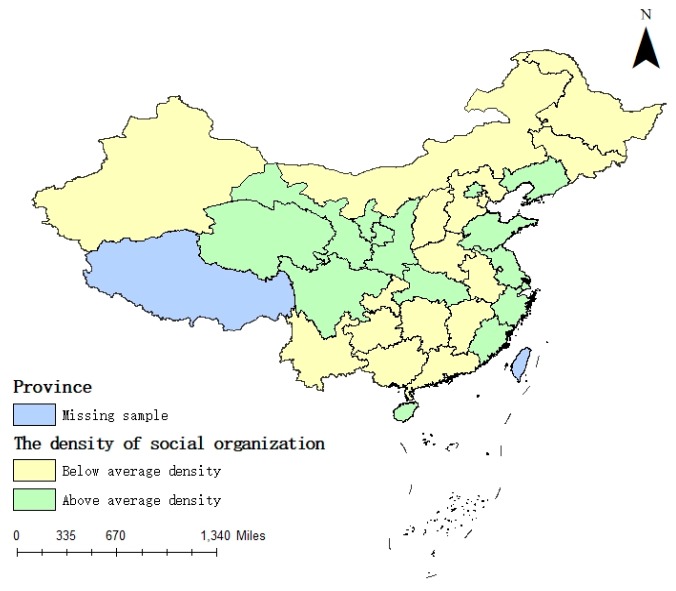
The density of social organizations.

**Figure 2:**
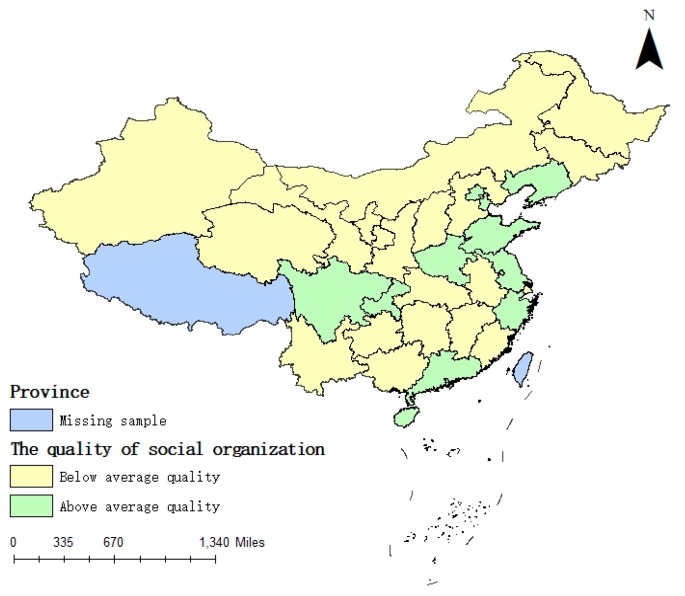
The quality of social organizations.

The average values of the density and quality of social organizations are 4.12 and 12.24% from 2007 to 2017, respectively. Then, the average values are adapted to visualize the density and quality of social organizations. As shown in Figure 1 and Figure 2, the degree of social organization density and social organization quality in the eastern coastal areas is higher than that in the inland regions. Both density and quality are above average in nearly all eastern coastal areas, suggesting that spatial heterogeneity exists in the density and quality of social organizations in China.

A number of studies have been conducted by institutions and scholars on government governance. The World Bank measures government governance by issuing World Governance Indicators (WGI), and the variables include voice and accountability, political stability and absence of violence/terrorism, government effectiveness, regulatory quality, rule of law and control of corruption [56]. The International Country Risk Guide (ICRG) dataset uses law and order, corruption, democratic accountability, bureaucracy quality, military in politics, and religion in politics to represent the dimensions of government governance [57]. In addition, many scholars use a single indicator, such as corruption control or financial status, to measure government governance [58,59].

Government governance in a broad sense includes not only self-governance but also the governance of economic activities, markets and social and public affairs [60]. This paper selects the indicators of business environment, administrative efficiency, public management and financial self-sufficiency to build an index of the government governance. Business environment is represented by the proportion of private enterprises and individual personnel in total urban employment; administrative efficiency is represented by the number of public officials per 10,000 RMB of financial revenue; and public management and financial self-sufficiency are measured by the general budget expenditure as a percentage of the regional GDP and the ratio of the general budget revenue to the general budget expenditure, respectively. As mentioned above, the entropy method is used to calculate the weight and then the weighted summation all of the variables to obtain the index system. Before using the entropy method, negative indicators are converted into positive values; administrative efficiency is the only negative indicator in this study. Therefore, we use the reciprocal method to make this indicator positive and then adopt the entropy method to calculate the weight of each indicator. The calculation process of the basic entropy method is set as (1)–(4).

In line with the studies investigating public service supply conducted by Wu and Li [61] and Tian et al. [62], this paper selects the two indicators of urbanization level and population density as control variables. The independent and control variables are shown in Table 2.

### 2.2. Model

First, this paper analyzes the relationship among social organization participation, government governance and the equalization of basic public services. Then, the benchmark model is decomposed to discuss the impact of social organization density and quality on the equalization of basic public services after controlling for government governance. Furthermore, to explore whether government governance can promote the equalization of basic public services by influencing the participation of social organizations, the mediation effect and linkage effect are considered. Finally, we discuss the heterogeneity in different basic public services based on social organization participation and government governance.

#### 2.2.1. Benchmark Model

To reflect the continuity and dynamic adjustment process of the equalization of basic public services, the first-order lag term of the equalization of basic public services is included in the regression model. Furthermore, to eliminate the influence of different dimensions, all variables are logarithmically processed. The benchmark model is set as shown in (5)–(6).

(5) $$\ln PS_{it}=\alpha_{0}+\beta_{1}\ln GO_{it}+\beta_{2}\ln SO_{it}+\beta_{3}\ln PS_{i,t-1}+\sum^{\text{​}}\beta_{j}X_{it}+u_{i}+\varepsilon_{it}$$

(6) $$\;\ln PS_{it}=\alpha_{0}+\beta_{1}\ln SD_{it}+\beta_{2}\ln SQ_{it}+\beta_{3}\ln GO_{it}+\beta_{4}\ln PS_{i,t-1}+\sum^{\text{​}}\beta_{j}X_{it}+u_{i}+\varepsilon_{it}\;$$

Model (5) analyzes the impact of social organization participation and government governance on the equalization of basic public services. Model (6) decomposes social organization participation into social organization density and social organization quality. We investigate the influence of social organization participation on the equalization of basic public services after controlling for the government governance. The subscripts *i* and *t* represent the year and province, respectively; *μ* is an unobservable regional effect, *ε* is a random disturbance term, *α* is a constant term, and *β* is a parameter to be estimated. *PS* is basic public service equalization, and *PS_i,t_*_−1_ is its first-order lag term. *GO* is a comprehensive indicator of the government governance, *SO* is the participation of social organizations, and *SD* and *SQ* represent the density and quality of social organizations, respectively. *X_it_* denotes a vector of control variables. After adding the first-order hysteresis of the dependent variable, the model is transformed into a dynamic panel model. If a general fixed effect or stochastic effect analysis is used, there may be bias in the estimation; therefore, we choose the system generalized method of moments (SYS-GMM), which is commonly adopted for dynamic panel models, for the benchmarking regression. The method can solve individual heterogeneity, missing variables and endogenous problems effectively and has better estimation efficiency. When using the SYS-GMM, sequence correlation tests and over-identification tests must be performed. We use the Sargan test to determine whether there are over-identifying restrictions.

#### 2.2.2. Mediation Effect Model

A mediation effect model can analyze how independent variables affect a dependent variable. To test whether the density and quality of social organizations are mediators of the equalization of basic public services, we construct a recursive model to test the mechanism and path of the equalization of basic public services by the government governance and the participation of social organizations. The mediation effect model proposed by Baron and Kenny [63] and the test procedure proposed by Wen and Ye [54] were used along with the SYS-GMM, and the model is set as shown in (7)–(9).

(7) $$\ln PS_{it}=\alpha_{0}+\beta_{1}\ln GO_{it}+\beta_{2}\ln PS_{i,t-1}+\sum^{\text{​}}\beta_{j}X_{it}+u_{i}+\varepsilon_{it}$$

(8) $$\;\ln Social_{it}=\alpha_{0}+\beta_{1}\ln GO_{it}+\beta_{2}\ln Social_{i,t-1}+\sum^{\text{​}}\beta_{j}X_{it}+u_{i}+\varepsilon_{it}\;$$

(9) $$\;\;\mathrm{Ln}PS_{it}=\alpha_{0}+\beta_{1}\ln GO_{it}+\beta_{2}\ln Social_{it}+\beta_{3}\ln PS_{i,t-1}+\sum^{\text{​}}\beta_{j}X_{it}+u_{i}+\varepsilon_{it}\;$$

*Social* in the model represents social organization density and social organization quality. Model (7) examines the impact of the government governance on the equalization of basic public services. Model (8) examines the impact of the government governance on social organization participation. Model (9) comprehensively examines the significance of the government governance and social organization participation in the equalization of basic public services. Models (8) and (9) are tested under the condition that Model (7) is satisfied, and if the variables of social organization participation and government governance are both significant, the coefficient of social organization participation in Model (9) is significant, indicating a significant partial mediation effect. If the government governance in Model (8) is significant, social organization participation in Model (9) is significant, but the government governance is not significant, indicating a complete mediation effect.

#### 2.2.3. Linkage Effect Model

We decompose government governance into the indicators of business environment, administrative efficiency, public management and financial self-sufficiency and then construct an interaction with social organization density and quality to further analyze the linkage among social organization participation, government governance and basic public services equalization. If the result of interaction is significant, the linkage effect exists. To avoid multiple collinearity, the independent variables are centralized to construct the model, which is set as shown in (10)–(11).

(10) $$\begin{array}{c} \ln PS_{it}=\alpha_{0}+\beta_{1}(\ln{SD}_{it}-\bar{lnSD_{it}})*\left({\ln\mathrm{Government}}_{it}-\bar{ln{\mathrm{Government}}_{it}}\right)+\beta_{2}\ln{\mathrm{SD}}_{it}\\ +\beta_{3}\ln{\mathrm{Government}}_{it}+\beta_{4}\ln PS_{i,t-1}+\sum^{\text{​}}\beta_{j}X_{it}+u_{i}+\varepsilon_{it} \end{array}$$

(11) $$\begin{array}{c} \ln PS_{it}=\alpha_{0}+\beta_{1}(\ln{\mathrm{SQ}}_{it}-\bar{lnSQ_{it}})*\left({\ln\mathrm{Government}}_{it}-\bar{ln{\mathrm{Government}}_{it}}\right)+\beta_{2}\ln{\mathrm{SQ}}_{it}\\ +\beta_{3}\ln{\mathrm{Government}}_{it}+\beta_{4}\ln PS_{i,t-1}+\sum^{\text{​}}\beta_{j}X_{it}+u_{i}+\varepsilon_{it} \end{array}$$

*Government* in the model represents the business environment, administrative efficiency, public management and financial self-sufficiency; $\;\bar{lnSD_{it}}$, $\bar{lnSQ_{it}}$ and $\bar{lnGovernment_{it}}$ mean the average of the $\ln SD_{it}$, $\;\ln SQ_{it}$ and ${\mathrm{lnGovernment}}_{it}$, respectively, and other indicators are consistent with the above models.

#### 2.2.4. Heterogeneity Analysis Model

To analyze the heterogeneous influences of social organization participation and government governance on different basic public services, we construct a heterogeneity model. The heterogeneity analysis model is similar to the linkage effect, and the core is the significance of the interaction between social organization participation and government governance. To avoid multiple collinearity, the independent variables are centralized to construct the model, which is set as shown in (12).

(12) $$\begin{array}{c} \ln Public_{it}=\alpha_{0}+\beta_{1}(\ln{\mathrm{SO}}_{it}-\bar{lnSO_{it}})*\left({\mathrm{lnGO}}_{it}-\bar{lnGO_{it}}\right)+\beta_{2}\ln{\mathrm{SO}}_{it}+\beta_{3}\ln{\mathrm{GO}}_{it}\\ +\beta_{4}\ln PS_{i,t-1}+\sum^{\text{​}}\beta_{j}X_{it}+u_{i}+\varepsilon_{it} \end{array}$$

*Public* represents the equalization levels of six basic public service indicators, $\bar{lnSO_{it}}\;$ and $\bar{lnGO_{it}}$ mean the average of ${\mathrm{lnSO}}_{it}$, ${\;\mathrm{lnGO}}_{it}$, respectively, and the other indicators are consistent with the above model.

### 2.3. Data Sources and Descriptive Statistics

**Figure 3:**
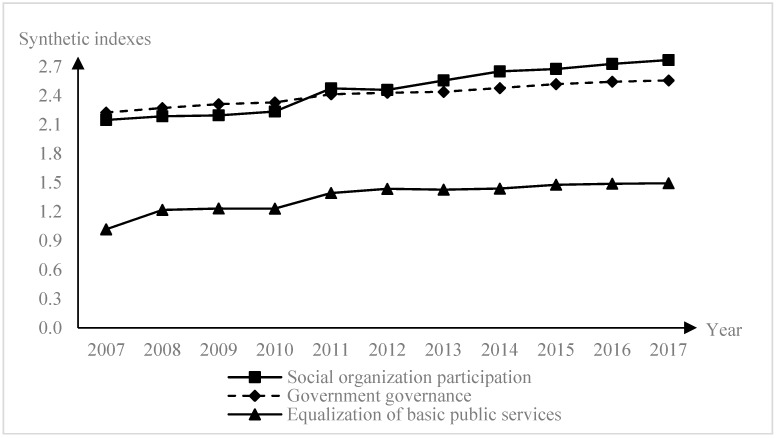
The synthetic index of variables.

This paper selects panel data from 30 provinces in mainland China from 2007 to 2017 (Tibet is excluded from the sample due to a lack of data). The main data sources are the *China Statistical Yearbook*, *China Civil Affairs Statistics Yearbook*, *China Social Organizations Yearbook, China Inspection Statistics Yearbook*, *Chinese Health Statistics Yearbook*, *China Science and Technology Statistical Yearbook*, *Education Statistical Yearbook in China*, *China Statistical Yearbook for Regional Economy*, *China Statistical Yearbook of the Tertiary Industry*, *China Population & Employment Statistics Yearbook*, *China Labor Statistics Yearbook* and the annual statistical bulletins and inspection bulletins of each province. The missing variables are supplemented by the interpolation method. The synthetic index of variables from 2007–2017 are shown in Figure 3, and the descriptive statistics of each variable after assuming the logarithm are presented in Table 3.

All of the three synthetic index variables in 2007–2017 present a slow growth trend. The synthetic index of social organizations’ participation and government governance is higher than the equalization of the basic public services index. An interesting phenomenon is that the synthetic index of social organization participation exceeds government governance after 2011, which may be related to the policy of social organizations outlined in “The Twelfth Five-year Plan of China”. The standard error of the equalization of basic public services, social organization participation and government governance are 0.018, 0.084 and 0.029, smaller than other variables, which means the variables are stable.

## 3. Results

### 3.1. Benchmark Regression Results

#### 3.1.1. Social Organization Participation and Government Governance Benchmark Regression

We gradually add the control variables to the benchmark regression. The results show that social organization participation and the government governance have a significant positive impact on the equalization of basic public services, but the regression coefficient of the government governance is far greater than that of social organization participation. These results are consistent with the reality that the supply of basic public services is a basic function of the government, which reflects the dominant and controlling role of the government in promoting the equalization of basic public services. These results also show that social organizations play a weaker role in promoting the equalization of basic public services and that their provision of basic public services is still in the early stage. On the one hand, this finding demonstrates the weakness, low coverage and small service population of social organizations in China, but on the other hand, it shows that some elements of social organizations restrict their impact on the equalization of basic public services. The benchmark regression results are shown in Table 4.

#### 3.1.2. Social Organization Participation Decomposition Regression

The benchmark regression results show that the participation of social organizations has a significant positive impact on the equalization of basic public services, but the degree of the influence is weak. To analyze how social organizations, influence the equalization of basic public services and determine the obstacles that restrict their efforts, we divided the participation of social organizations into density and quality sub-indicators for further analysis.

First, we find that the participation of social organizations has a significant positive impact on promoting the equalization of basic public services without controlling for the government governance as its coefficient is slightly higher than that obtained after controlling for the government governance, which verifies the benchmark regression results. Then, Columns (2) and (3) decompose the participation of social organizations. After controlling for government governance, increasing the social organization density is found to significantly promote the equalization of basic public services, while improving the social organization quality has a negative but nonsignificant impact on the equalization of basic public services. The primary way for social organizations to participate in the equalization of basic public services in China is to expand the population coverage that they can service. Currently, the quality of social organizations has no driving effect on the equalization of basic public services. Column (4) shows the results obtained by including both the density and quality of social organizations in the model and reveals that the density of social organizations still has a significant positive impact, while the quality has a negative but nonsignificant impact. This finding further proves that the main way for social organizations to participate in the equalization of basic public services in China is to expand the number of suppliers rather than improve the quality of that suppliers and that the participation of social organizations in basic public services has characteristics of extensive development. The decomposition regression results are shown in Table 5.

### 3.2. Mediation Effect Analysis

Through the mediation effect test, we analyze whether social organization participation can mediate the equalization of basic public services. The regression results show that the regression coefficient of the government governance is significantly positive in all models, suggesting that the government governance plays a leading role in promoting the equalization of basic public services. Improving the governance capacity, such as the business environment, administrative efficiency and public management ability, can improve the equalization of basic public services. Brackets (4) and (5) report the regression results of the dependent variables based on the independent and mediator variables. After adding social organization density as a mediator variable, the coefficient of the government governance decreases significantly, and the coefficient of social organization density is significantly positive. However, when social organization quality is used as a mediator variable, the regression coefficient of the government governance does not decrease. This finding indicates that the increase in the density of social organizations has a mediation effect on promoting the equalization of basic public services and that improving social organization quality does not currently have a mediation effect. In the process of basic public services equalization, the effect of social organization participation is manifested mainly through increased density, and the establishment of a comprehensive network of social organizations remains a powerful way to promote the equalization of basic public services. The mediation effect regression results are shown in Table 6.

### 3.3. Linkage Effect Analysis

After conducting the mediation effect test, the interaction between the four sub-indexes of the government governance and the participation of social organizations is constructed to examine the linkage effect on the equalization of basic public services. The self-correlation test cannot be passed when only the first-order hysteresis is added to the model; therefore, to solve the autocorrelation problem of the disturbance item, the second-order hysteresis of the dependent variable is introduced [64] (p. 297). The interaction results between government governance and social organization density are presented in Table 7.

The results show that the interaction between the sub-indexes of the government governance and the density of social organizations are all significant, and improvement in the government governance could lead to an increase in social organization density and then have a positive impact on the equalization of basic public services. This result not only emphasizes the role of social organization density in the equalization of basic public services but also highlights its significant mediation effect. The results also show that the linkage effect between social organization density and the business environment is the most obvious, and as an important manifestation of the internal governance of the government, administrative efficiency and social organization density also have a significant linkage effect. This finding shows that the transformation of government function in China has contributed to building a good business environment, increasing the administrative efficiency, and thereby stimulating the vitality of social organizations, thus promoting the equalization level of basic public services. In addition, the direct purchase of services by the government is an important way for social organizations to participate in the equalization of basic public services. The positive linkage effect of public management, financial self-sufficiency and social organization density verifies that the increasing number and variety of government purchases drive the increase in the density of social organizations, thereby promoting the equalization of basic public services.

We then analyze the interaction between the four sub-indices of the government governance and the quality of social organizations. The results show that the business environment, public management and social organization quality have a positive linkage effect. The interactions among administrative efficiency, financial self-sufficiency and social organization quality are significantly negative, inhibiting the development of basic public services equalization to a certain extent. This result partially reveals why social organization quality does not have a mediation effect on promoting the development of basic public services. With improvement in the business environment and public management investment, the market access threshold is gradually reduced. The field of basic public services supply continues to expand, and the demand for high-quality staff in social organizations is increasing. The importance of improving the quality of staff to provide more refined and high-quality service has become a social consensus. Therefore, the business environment, public management and social organization density have a positive linkage effect. Administrative efficiency, financial self-sufficiency and social organization quality are the concentrated characteristics of internal governance capabilities; therefore, there is a weak possibility of a linkage effect in theory. In addition, governments with a higher administrative efficiency and higher financial self-sufficiency often have the ability to supply high-quality basic public services and, to a certain extent, limit the participation of social organizations. In the sample, two-thirds of the provinces have lower quality than average (Figure 2), but for the density, the number is approximately one-half (Figure 1). Moreover, the interaction between government governance and the density of social organizations is significantly positive, but the interaction among government efficiency, financial self-sufficiency and social organization quality are negatively significant, with coefficients of −0.01 and −0.069, but the coefficients of density are 0.010 and 0.018. The weak linkage between the government governance and social organization quality indicates low investment and limited attention to improving the quality of social organizations. One reason explaining this finding is the absence of a comprehensive social organization network; thus, the government pays more attention to the increase in the density of social organizations to the detriment of their quality improvement. This finding also reflects the problem of weak financial support from the government for the training of high-quality staff in social organizations. The results of the interaction between the government governance and social organization quality are shown in Table 8.

### 3.4. Heterogeneity Analysis

The results of the heterogeneity analysis show that the interaction between government governance and social organization participation has a positive impact on medical and health care, public education, environmental protection and public culture but nonsignificant impact on public science and social welfare. The interaction between medical and health care and environmental protection show that with the improving of government governance, the effect of social organizations participation become more obvious. In contrast, the influence of public culture shows that the participation of social organizations has stronger driving ability than government governance. Although the interaction of basic public education is significant, the linkage effect of social organizations participation and government governance is weaker than others. This finding also suggests that the linkage effect between social organizations and the government governance on public science and social welfare is weak, which hinder them from forming synergy in supplying these services. Improvement in the supply level of these two basic public services, especially public science, is difficult to achieve by relying on an increase in the density of social organizations. Instead, such improvement requires a large number of high-quality employees. This issue also reflects the current problem that the quality of social organizations limits the ability of such organizations to drive the equalization of basic public services in China. The results of the heterogeneity analysis are shown in Table 9.

## 4. Discussion

In this section, we performed some auxiliary tests to ensure the robustness of the conclusions of this paper. This paper conducts a robustness analysis in three aspects. First, we adopt the method of replacing indicators. The participation of social organizations is measured by the scale of social organizations (the number of employees in social organizations at the end of the year), and the government governance is measured by corruption control indicators. The regression results show that the participation of social organizations and the government governance have a significant positive impact on the equalization level of basic public services regardless of whether the control variables are added. The regression coefficient of the government governance measured by corruption control has decreased compared with that in the benchmark regression, but it still has a significant positive effect on the equalization of basic public services at the 1% level of significance. The results obtained by replacing the indicators are shown in Table 10.

The social organizations are then divided into private non-enterprises and social groups (the number of foundations is small; thus, they are not considered). The regression results show that private non-enterprise participation and the government governance have a significant positive impact on the equalization of basic public services regardless of whether other control variables are added, while social group participation does not. We believe the reason is that private non-enterprises mainly undertake the functions of providing social public services, and social groups play a more important role in promoting communication and development within an industry in China. After decomposing social organization participation into social organization density and quality, an increase in density and improvement in the quality of private non-enterprises are found to have a significant positive impact on the development of basic public services equalization, but quality has less of an influence than density in terms of the significance level and the regression coefficient. An increase in social group density has a significant impact on the equalization of basic public services, but the driving ability of quality improvement is not significant. This result shows that the function of social groups limits their driving role in the equalization of basic public services and that social organization quality has a weaker ability to drive basic public services equalization than social organization density. The results of the grouped regression are shown in Table 11.

Finally, the Bootstrap method is used to test the mediation effect. If the confidence interval does not contain 0, a significant mediation effect exists. Here, the mediator variables include the number and scale of social organizations, and we set government governance as the key independent variable. The results show that both direct and indirect effects exist in the 95% confidence interval of both the number and scale of social organizations, indicating that a significant mediation effect exists. The results of the bootstrap method are shown in Table 12.

## 5. Conclusions

This paper empirically explores the relationship among social organization participation, government governance and the equalization of basic public services, and the following conclusions are obtained. (1) The participation of social organizations and improvement in the government governance can significantly promote the equalization of basic public services, and social organization density plays a stronger role than social organization quality. (2) Social organization density is a mediator variable in the effect of the governance capacity promoting the equalization level of basic public services. (3) The linkage effects between indicators of the government governance and social organization participation are heterogeneous, and the linkage effect with social organization density is the strongest. (4) The interaction between the government governance and social organization participation has a positive impact on medical and health care, public education, environmental protection and public culture but a negative and nonsignificant impact on public science and social welfare.

The results of this paper show that encouraging the participation of social organizations is of great significance for achieving the equalization of basic public services in China. To alleviate the pressure of government on the equalization of basic public services, the mediator role of social organizations should be played, especially in the aspects of medical and health care, environmental protection, public culture and public education. However, the participation of social organizations remains inadequate. It is necessary to support the set of social organizations and increase their density and coverage. To promote the development of high-quality basic public services, the existing social organizations should optimize the age structure and academic structure of their employees and the professional quality and strive to achieve precise and professional supply goals. Moreover, to form synergy between the government and social organizations for the equalization of basic public services, the government should construct a good business environment and improve its own administrative efficiency. It is also necessary to allocate fiscal funds efficiently and reasonably, increase financial self-sufficiency, and accelerate the long-term participation of social organizations in the equalization of basic public services by improving the government governance capacity.

## Figures and Tables

**Table 1:** Dependent variables and weights.

Variable Name	Variable Category	Indicator	Weight
Equalization of basic public services	Public Education	Number of primary school students per 100,000 people	0.001
		Number of middle school students per 100,000 people	0.035
		Number of high school students per 100,000 people	0.029
	Medical and Health Care	Number of hospital beds per 1000 people	0.098
		Number of health institutions per 10,000 people	0.059
		Number of health personnel per 10,000 people	0.058
	Public Culture	Number of public libraries	0.058
		Per capita possession of public library holdings	0.218
		Broadcast integrated population coverage	0.025
	Public Science	Number of invention patents per 100,000 people	0.119
		Harmless treatment rate of domestic garbage	0.041
		Urban sewage treatment rate	0.027
	Environmental Protection	Lake area per 100,000 people	0.075
		Number of nature reserves	0.037
		Number of parks	0.044
	Social Welfare	Number of beds in child adoption institutions	0.023
		Number of elderly activity stations/rooms/centers	0.053

**Table 2:** Independent and control variables and weights.

Variables Name	Variable Category	Indicator	Weight
Social organization participation	Social organization density	Number of social organizations per 10,000 people	0.516
	Social organization quality	Proportion of undergraduate workers in social organizations	0.484
Government governance	Business environment	Proportion of private enterprises and individual personnel in urban total employment	0.359
	Administrative efficiency	Number of public officials for the financial revenue per 10,000 RMB	0.258
	Public management	General budget expenditure as a percentage of regional GDP	0.262
	Financial self-sufficiency	Ratio of general budget revenue to general budget expenditure	0.121
Population density		Urban population density	
Urbanization level		Urbanization rate	

**Table 3:** Descriptive statistics.

Variables Name	Observations	Average	Standard Error	Minimum	Maximum
Equalization of basic public services	330	0.128	0.018	0.079	0.174
Social organization participation	330	0.389	0.084	0.173	0.596
Government governance	330	0.382	0.029	0.308	0.468
Social organization quality	330	0.983	0.284	0.121	1.760
Social organization density	330	0.586	0.156	0.194	1.035
Business environment	330	1.644	0.093	1.291	1.841
Administrative efficiency	330	1.506	0.320	0.744	2.497
Public management	330	1.324	0.169	0.940	1.797
Financial self-sufficiency	330	1.680	0.170	1.170	1.978
Population density	330	3.403	0.195	2.793	3.776
Urbanization level	330	1.720	0.103	1.450	1.952

**Table 4:** Benchmark regression results.

Variables	(1)	(2)	(3)
Government governance	0.131 ***	0.153 ***	0.156 ***
	(0.012)	(0.010)	(0.012)
Social organization participation	0.008 ***	0.010 ***	0.011 ***
	(0.003)	(0.004)	(0.004)
Urbanization level		−0.015 ***	−0.016 ***
		(0.003)	(0.004)
Population density			0.004
			(0.003)
Constant	0.001 (0.002)	0.018 ***	0.004
		(0.004)	(0.012)
Lag (1)	0.603 *** (0.006)	0.607 ***	0.599 ***
		(0.005)	(0.012)
AR (1)	0.000	0.000	0.000
AR (2)	0.899	0.733	0.775
Sargan	0.230	0.239	0.235

Notes: Standard errors are shown in parentheses; *** denotes significance at the 1% levels.

**Table 5:** Social organization participation decomposition regression.

Variables	(1)	(2)	(3)	(4)
Social organization participation	0.037 ***			
	(0.003)			
Social organization density		0.033 ***		0.033 ***
		(0.002)		(0.001)
Social organization quality			−0.001	−0.001
			(0.001)	(0.001)
Government governance		0.079 ***	0.033 ***	0.084 ***
		(0.011)	(0.001)	(0.011)
Urbanization level	0.003	−0.014 ***	−0.013 ***	−0.013 ***
	(0.001)	(0.003)	(0.003)	(0.003)
Population density	0.009 ***	0.011 **	0.008 **	0.011 ***
	(0.002)	(0.004)	(0.003)	(0.003)
Lag (1)	0.667 ***	0.533 ***	0.568 ***	0.531 ***
	(0.008)	(0.009)	(0.011)	(0.010)
Constant	−0.001	0.002	−0.020	−0.003
	(0.009)	(0.014)	(0.013)	(0.013)
AR (1)	0.000	0.000	0.000	0.000
AR (2)	0.149	0.090	0.654	0.099
Sargan	0.278	0.300	0.272	0.295

Notes: Standard errors are shown in parentheses; *, **, and *** denote significance at the 10%, 5%, and 1% levels, respectively.

**Table 6:** Mediation effect analysis results.

Variables	PS	SD	SQ	PS	
	(1)	(2)	(3)	(4)	(5)
Government governance	0.170 ***	0.072 ***	0.260	0.068 ***	0.177 ***
	(0.010)	(0.003)	(0.214)	(0.011)	(0.010)
Social organization density				0.031 ***	
				(0.002)	
Social organization quality					−0.002 *
					(0.001)
Urbanization level	−0.011 ***	−0.222 ***	0.619 ***	−0.015 ***	−0.012 ***
	(0.002)	(0.019)	(0.141)	(0.003)	(0.003)
Population density	0.003	0.072 ***	−0.068	0.008 *	0.004
	(0.003)	(0.003)	(0.063)	(0.005)	(0.003)
Lag (1)	0.608 ***	1.013 ***	0.580 ***	0.560 ***	0.607 ***
	(0.012)	(0.005)	(0.030)	(0.009)	(0.010)
Constant	0.002	−0.067 ***	−0.492 **	0.003	−0.004
	(0.012)	(0.026)	(0.215)	(0.019)	(0.015)
AR (1)	0.000	0.065	0.003	0.000	0.000
AR (2)	0.930	0.748	0.771	0.079	0.913
Sargan	0.233	0.298	0.394	0.246	0.239

Notes: Standard errors are shown in parentheses; *, **, and *** denote significance at the 10%, 5%, and 1% levels, respectively.

**Table 7:** Interaction between the government governance and social organization density.

Variables	(1)	(2)	(3)	(4)	(5)
Business environment × Social organization density	0.031 **				0.105 **
	(0.012)				(0.047)
Public management × Social organization density		0.030 ***			0.041 **
		(0.011)			(0.021)
Government efficiency × Social organization density			0.010 **		0.034**
			(0.004)		(0.016)
Financial self-sufficiency × Social organization density				0.018 *	0.018
				(0.009)	(0.034)
Social organization density	0.024 ***	0.025 ***	0.022 ***	0.014 ***	0.026 ***
	(0.002)	(0.002)	(0.002)	(0.002)	(0.004)
Business environment	0.010 ***				0.006 **
	(0.003)				(0.005)
Public management		0.015 ***			−0.013
		(0.004)			(0.008)
Government efficiency			−0.011 ***		−0.010 **
			(0.001)		(0.005)
Financial self-sufficiency				−0.022 ***	−0.013 *
				(0.003)	(0.008)
Urbanization level	−0.013 ***	0.015 ***	−0.014 ***	0.019 ***	−0.013 ***
	(0.003)	(0.004)	(0.004)	(0.006)	(0.003)
Population density	0.013 ***	0.016 ***	0.014 ***	0.016 ***	0.013 ***
	(0.002)	(0.002)	(0.002)	(0.002)	(0.002)
Lag (1)	0.502 ***	0.495 ***	0.509 ***	0.471 ***	0.511 ***
	(0.012)	(0.008)	(0.007)	(0.008)	(0.020)
Lag (2)	0.119 ***	0.124 ***	0.159 ***	0.121 ***	0.170 ***
	(0.010)	(0.011)	(0.008)	(0.008)	(0.017)
Constant	0.035 ***	−0.026 **	0.021 **	−0.003	0.035 ***
	(0.008)	(0.010)	(0.008)	(0.009)	(0.000)
AR (1)	0.000	0.000	0.000	0.000	0.000
AR (2)	0.271	0.191	0.285	0.289	0.625
Sargan	1.000	1.000	1.000	1.000	1.000

Notes: Standard errors are shown in parentheses; *, **, and *** denote significance at the 10%, 5%, and 1% levels, respectively.

**Table 8:** Interaction between the government governance and social organization quality.

Variables	(1)	(2)	(3)	(4)	(5)
Business environment × Social organization quality	0.086 ***				0.048 ***
	(0.011)				(0.015)
Public management × Social organization quality		0.043 ***			0.035 *
		(0.009)			(0.018)
Administrative efficiency × Social organization quality			−0.010 ***		−0.005
			(0.002)		(0.008)
Financial self-sufficiency × Social organization quality				−0.069 ***	−0.077 ***
				(0.004)	(0.021)
Social organization quality	−0.011 ***	−0.004 ***	−0.010 ***	−0.007 ***	−0.012 ***
	(0.001)	(0.001)	(0.001)	(0.001)	(0.001)
Business environment	0.077 ***				0.064 ***
	(0.012)				(0.017)
Public management		0.024 *			−0.015
		(0.013)			(0.022)
Administrative efficiency			0.009 ***		0.023 *
			(0.003)		(0.013)
Financial self-sufficiency				0.040 ***	−0.003
				(0.005)	(0.018)
Urbanization level	0.014 ***	0.007 **	0.014 ***	0.061 ***	0.058 ***
	(0.004)	(0.003)	(0.005)	(0.004)	(0.009)
Population density	0.014 ***	0.006 ***	0.008 ***	0.012 ***	0.013 ***
	(0.002)	(0.002)	(0.002)	(0.003)	(0.004)
Lag (1)	0.476 ***	0.554 ***	0.534 ***	0.479 ***	0.413 ***
	(0.015)	(0.010)	(0.011)	(0.011)	(0.032)
Lag (2)	0.199 ***	0.162 ***	0.140 ***	0.115 ***	0.103 ***
	(0.011)	(0.009)	(0.012)	(0.007)	(0.026)
Constant	0.113 ***	0.002	0.033 *	−0.137 ***	−0.025
	(0.023)	(0.007)	(0.018)	(0.017)	(0.069)
AR (1)	0.000	0.000	0.000	0.000	0.003
AR (2)	0.643	0.811	0.480	0.464	0.102
Sargan	1.000	1.000	1.000	1.000	1.000

Notes: Standard errors are shown in parentheses; *, **, and *** denote significance at the 10%, 5%, and 1% levels, respectively.

**Table 9:** Basic public service equalization heterogeneity analysis.

Variables	Medical and Health Care	Public Education	Environmental Protection	Public Science	Public Culture	Social Welfare
Government governance × Social organization participation	5.691 ***	0.441 **	4.273 ***	0.492	5.243 ***	−2.177
	(1.736)	(0.177)	(1.024)	(0.333)	(0.209)	(1.395)
Government governance	5.006 ***	0.079 ***	1.365 ***	0.809 ***	−0.404 ***	0.596 ***
	(0.247)	(0.017)	(0.159)	(0.050)	(0.055)	(0.094)
Social organization participation	−0.113	−0.022 ***	−0.451 ***	0.086 ***	0.074 ***	−0.147 ***
	(0.103)	(0.005)	(0.038)	(0.013)	(0.023)	(0.027)
Urbanization level	−1.242 ***	−0.110 ***	−1.265 ***	0.235 ***	0.215 ***	−0.352 ***
	(0.079)	(0.007)	(0.082)	(0.026)	(0.031)	(0.053)
Population density	−0.217 ***	−0.025 ***	0.214 ***	−0.058 ***	0.027 ***	0.074 ***
	(0.074)	(0.003)	(0.046)	(0.016)	(0.007)	(0.024)
Lag (1)	0.664 ***	0.768 ***	0.792 ***	0.436 ***	0.926 ***	0.825 ***
	(0.006)	(0.011)	(0.006)	(0.022)	(0.004)	(0.015)
Constant	1.514 ***	0.545 ***	1.491 ***	−0.328 ***	−0.588 ***	0.516 ***
	(0.293)	(0.022)	(0.232)	(0.080)	(0.057)	(0.075)
AR (1)	0.000	0.000	0.024	0.002	0.000	0.003
AR (2)	0.096	0.755	0.376	0.217	0.104	0.881
Sargan	0.253	0.231	0.362	0.230	0.799	0.545

Notes: Standard errors are shown in parentheses; *, **, and *** denote significance at the 10%, 5%, and 1% levels, respectively.

**Table 10:** Robustness test by replacing the indicators.

Variables	Replacing Social Organization Participation Variables		Replacing Government Governance Variable	
Government governance	0.147 *** (0.007)	0.148 *** (0.007)		
Social organization participation			0.031 ***	0.030 ***
			(0.002)	(0.002)
Corruption control			0.003 ***	0.005 **
			(0.001)	(0.001)
Social organization scale	0.012 ***	0.013 ***		
	(0.001)	(0.002)		
Urbanization level		0.003		0.001 **
		(0.004)		(0.001)
Population density		0.008 **		0.003 **
		(0.003)		(0.002)
Lag (1)	0.549 ***	0.534 ***	0.707 ***	0.708 ***
	(0.010)	(0.012)	(0.004)	(0.006)
Constant	−0.056 ***	−0.093 ***	0.025 ***	0.032 ***
	(0.006)	(0.018)	(0.002)	(0.001)
AR (1)	0.000	0.000	0.000	0.000
AR (2)	0.946	0.897	0.140	0.112
Sargan	0.231	0.266	0.230	0.241

Notes: Standard errors are shown in parentheses; *, **, and *** denote significance at the 10%, 5%, and 1% levels, respectively.

**Table 11:** Robustness test by social organization group.

Variables	Private Non-Enterprises			Social Groups		
	(1)	(2)	(3)	(1)	(2)	(3)
Social organization participation	0.033 ***	0.034 ***		0.004	0.006	
	(0.004)	(0.004)		(0.003)	(0.005)	
Government governance	0.106 ***	0.124 ***	0.066 ***	0.161 ***	0.165 ***	0.096 ***
	(0.012)	(0.017)	(0.012)	(0.006)	(0.010)	(0.010)
Social organization density			0.025 ***			0.033 ***
			(0.001)			(0.003)
Social organization quality			0.001*			−0.002
			(0.001)			(0.001)
Urbanization level		−0.021 ***	−0.029 ***		−0.015 ***	0.008 ***
		(0.002)	(0.002)		(0.003)	(0.002)
Population density		0.004	0.006		0.004	0.007 *
		(0.002)	(0.004)		(0.003)	(0.004)
Lag (1)	0.555 ***	0.584 ***	0.559 ***	0.571 ***	0.602 ***	0.551 ***
	(0.005)	(0.006)	(0.007)	(0.003)	(0.013)	(0.012)
Constant	0.008 **	0.019 *	0.059 ***	−0.005 ***	0.001	−0.023 *
	(0.003)	(0.010)	(0.012)	(0.001)	(0.013)	(0.013)
AR (1)	0.000	0.000	0.000	0.000	0.000	0.000
AR (2)	0.364	0.427	0.053	0.896	0.993	0.271
Sargan	0.277	0.235	0.236	0.272	0.233	0.244

Notes: Standard errors are shown in parentheses; *, **, and *** denote significance at the 10%, 5%, and 1% levels, respectively.

**Table 12:** Robustness test of the mediation effect.

Variables	Mediation Effect	Coefficient	Standard Error	Z	P > Z	95% Confidence Interval
Social organization number	Indirect effect	0.039 ***	0.010	1.820	0.004	(0.001, 0.036)
	Direct effect	0.242 ***	0.037	8.280	0.000	(0.233, 0.378)
Social organization social	Indirect effect	0.019 **	0.010	1.860	0.030	(0.001, 0.041)
	Direct effect	0.303 ***	0.038	7.970	0.000	(0.229, 0.378)

Notes: ** and *** denote significance at the 5% and 1% levels, respectively.

## References

[1] China State Council Notice on Promoting the Equalization Plan of Basic Public Services during the 13th Five-Year Plan. http://www.gov.cn/zhengce/content/2017−03/01/content_5172013.htm.

[2] Wang P., Salamon L.M. (2010). Outsourcing Government-Financed Social Services to Civil Society Organizations: Lessons from China and Abroad.

[3] Teng S. (2003). Public governance theory and the changes. J. Chin. Aca. Gov..

[4] Zeng Z. (2006). Governance in public administration: Analysis of the concept of public governance. Chongqing Soc. Sci..

[5] Hill C.J., Lynn L.E. (2005). Is hierarchical governance in decline? Evidence from empirical research. J. Public Adm. Res. Theory.

[6] Rosenau J.N., Czempiel E.O. (2001). Governance without Government.

[7] Lan Z., Chen G. (2007). Notes on contemporary public administration theories in the west. J. Public Man..

[8] Rhodes R.A. (1996). The New Governance: Governing without Government1. Political Stu..

[9] Sorensen E.M. (2006). The changing role of politicians in processes of democratic governance. Am. Rev. Public Adm..

[10] Agranoff R. (2006). Inside collaborative networks: Ten lessons for public managers. Public Admin. Rev..

[11] Shi C. (2015). Research on the Government and Social Organization Cooperative Governance Performance Evaluation. Chin. Public Admin..

[12] Salamon L.M. (1995). Partners in Public Service: Government-Nonprofit Relations in the Modern Welfare State. Political Sci. Q..

[13] Besley T., Ghatak M. (2001). Government versus private ownership of public goods. Q. J. Econ..

[14] Schmitz P.W. (2015). Government versus private ownership of public goods: The role of bargaining frictions. J. Public Econ..

[15] Guiso L., Sapienza P., Zingales L. (2016). Long-term persistence. J. Eur. Econ. Assoc..

[16] Markussen T. (2011). Democracy, redistributive taxation and the private provision of public goods. Eur. J. Political Econ..

[17] Chen J.N. (2012). Contributions of environmental NGO to environmental education in China. IERI Procedia.

[18] Li L.Z., Zhang J.K., Shi D., Wang X. (2014). Research on the model construction of social organizations offering community-based home care for senior citizens. Mod. Urban Res..

[19] Vatn A. (2018). Environmental governance–From public to private?. Ecol. Econ..

[20] Zhao F., Hämäläinen J., Chen Y.T. (2017). Medical social work practice in child protection in China: A multiple case study in Shanghai hospitals. Soc. Work Health Care.

[21] Wei Y., Zhang H., Li X. (2018). Youth and teenager health service in community organizing: Prevention health care in China. Int. J. Adolesc. Youth.

[22] Liang Y., Liang H., Corazzini K.N. (2019). Predictors and patterns of home health care utilization among older adults in Shanghai, China. Home Health Care Serv. Q..

[23] Huang Y. (2019). Practice and consideration of social forces participating in the public libraries construction —Taking the Jiading district publiclibrary of Shanghai municipality as an example. Libr. Work Study.

[24] Hsu J.Y.J., Hasmath R. (2014). The local corporatist state and NGO relations in China. J. Contemp. China.

[25] Cai Y.S., Zhang J. (2016). Niche, connections and NGO operation in China. J. Chin. Gov..

[26] Taylor B., Campbell B. (2011). Quality, risk and governance: Social workers’ perspectives. Int. J. Leadersh. Public Serv..

[27] Xiong Y., Wang S. (2007). Development of social work education in china in the context of new policy initiatives: Issues and challenges. Soc. Work Educ..

[28] Li Y. (2012). The development of social organizations in China. China J. Soc. Work.

[29] Zhang J., Gao Y., Fu Y., Zhang H. (2007). Why does China enjoy so much better physical infrastructure?. Econ. Res. J..

[30] Krueger A.O. (1974). The political economy of the rent-seeking society. Am. Econ. Rev..

[31] Mauro P. (1998). Corruption and the composition of government expenditure. J. Public Econ..

[32] Fu Y., Zhang Y. (2007). Decentralization and fiscal expenditure structural deviation in China: The cost of competition for growth. Manag. World..

[33] Xu G., Wu Y. (2016). Basic public services and the restructuring of the public finance system in China: Retrospect and prospect. Asian Educ. Dev. Stud..

[34] Zhu C., Wu C. (2016). Public service motivation and organizational performance in Chinese provincial governments. Chin. Manag. Stud..

[35] Stoker G. (1998). Governance as theory: Five propositions. Int. Soc. Sci. J..

[36] Wang J. (2011). From administrative erosion to absorption and efficiency: The role of government in rural social management innovation. Marx. Real..

[37] Wichman C.J. (2016). Incentives, green preferences, and private provision of impure public goods. J. Environ. Econ. Manag..

[38] Dai X., Wu Y., Di Y., Li Q. (2009). Government regulation and associated innovations in building energy-efficiency supervisory systems for large-scale public buildings in a market economy. Energy Policy.

[39] Bai X.Y., Muhammad I.H., Li F.S., Gu Y.H., Muhammad S.H. (2017). An empirical study on application and efficiency of gridded management in public service supply of Chinese government. J. Sci. Technol. Policy Manag..

[40] Petrick M., Gramzow A. (2012). Harnessing communities, markets and the state for public goods provision: Evidence from post-socialist rural Poland. World Dev..

[41] Gupta D., Koontz T.M. (2019). Working together? Synergies in government and NGO roles for community forestry in the Indian Himalayas. World Dev..

[42] Knox C., Qun Z. (2007). Building public service-oriented government in China. Int. J. Public Sect. Manag..

[43] Yang G., Ma Y., Xue Y., Meng X. (2019). Does the Development of a High-Speed Railway improve the equalization of medical and health services? Evidence from China. Inter. J. Environ. Res. Public Health.

[44] Warner M., Hefetz A. (2002). Applying market solutions to public services: An assessment of efficiency, equity, and voice. Urban A Rev..

[45] Gong F., Lu H. (2009). Public expenditure structure, preference matching and fiscal decentralization. Manag. World.

[46] Li B., Li T., Yu M., Chen B. (2017). Can equalization of public services narrow the regional disparities in China? A spatial econometrics approach. China Econ. Rev..

[47] China State Council The 12th Five-year Plan of Basic Public Service System. http://www.gov.cn/zwgk/2012-07/20/content_2187242.htm.

[48] Xu J. (2019). Government’s experience in purchasing public technology services and its enlightenment to Guizhou Province. Eco. Res. Guide.

[49] Wang Y. (2012). Empirical research on equality of public environmental services and evaluation indicators. China Pop. Res. Env..

[50] Lucy W.H., Gilbert D., Birkhead G.S. (1977). Equity in local service distribution. Public Adm. Rev..

[51] Chen H. (2007). Research on basic public service system of China. Sci. Soc..

[52] Guo X.G. (1998). Application of improved entropy method in evaluation of economic result. Syst. Eng. Theory Pract..

[53] Archambault E. (2017). The Evolution of Public Service Provision by the Third Sector in France. Political Q..

[54] Liu B. (2015). Social organizations undertake public service research: Mechanisms, paths and norms. Theory Mon..

[55] Jing Y., Hu Y. (2017). From service contracting to collaborative governance: Evolution of government–nonprofit relations. Public Admin. Dev..

[56] Kaufmann D., Kraay A. (2007). Governance indicators: Where are we, where should we be going?. World Bank Res. Obs..

[57] Dong H., Yi K., Shu W., Lin C. (2018). Heterogeneity in the effects of government size and governance on economic growth. Eco. Mod..

[58] Graycar A. (2014). Awareness of corruption in the community and public Service: A victorian study. Aust. J. Public Admin..

[59] Cepparulo A., Giuriato L. (2016). Responses to global challenges: Trends in aid-financed global public goods. Dev. Policy Rev..

[60] Wang P. (2014). The inherent meaning and interrelationship of state governance, government administration and social governance. J. Chin. Acad. Gov..

[61] Wu Q., Li Y. (2010). Fiscal decentralization, competition and local land revenue. Financ. Trade Econ..

[62] Tian C.H., Li M.K., Li S.Q. (2014). Land fiscal and local public goods provision: Evidences from cities. J. Public Manag..

[63] Wen Z., Ye B. (2014). Analyses of mediating effects: The development of methods and models. Adv. Psychol. Sci..

[64] Chen Q. (2014). Advanced Econometrics and Stata Application.

